# Reviewed article: Nogueira HR, Amorim EL, Genaro GD, Lourenço MCS, Dantas VHS, Sousa LVA. Disparities in time-to-treatment initiation and therapeutic modalities for malignant melanoma of the skin: a cross-sectional study, Brazil, 2013-2023. Epidemiol Serv Saude. 2026:35;e20250025

**DOI:** 10.1590/S2237-96222026v35e20250025.a

**Published:** 2026-02-06

**Authors:** Cristiane Rocha Magalhães

**Affiliations:** 1 Instituto Nacional de Câncer, Rio de Janeiro, RJ, Brazil Instituto Nacional de Câncer Rio de Janeiro RJ Brazil

## Peer review A

## Questionnaire

Does the manuscript contain new and significant information to justify publication? Yes

Does the Abstract (Summary) clearly and accurately describe the content of the article? No

Is the problem significant and concisely stated? Yes

Are the methods described comprehensively? No

Are the interpretations and conclusions justified by the results? No

Is adequate reference made to other work in the field? Yes

## Manuscript Structure

Length of article is: Adequate

Number of tables is: Too few

Number of figures is: Adequate

Please state any conflict(s) of interest that you have in relation to the review of this paper. None

Interest: Good 

Quality: Good

Originality: Average

Overall: Good

Recommendation: Minor Revision

## Comments

O estudo aborda um tema de grande interesse na Saúde Coletiva e Epidemiologia, analisando disparidades regionais no acesso ao tratamento do melanoma maligno da pele no Brasil. A relevância está bem fundamentada na introdução mostrando o aumento da incidência de melanoma globalmente e no Brasil.

O manuscrito apresenta uma estrutura bem organizada. Porém, algumas partes do texto apresentam repetições (principalmente as frases sobre a legislação). Pequenos ajustes na redação poderiam melhorar a fluidez da leitura, evitando frases longas e complexas, tornando o texto mais atrativo ao leitor.

Na introdução, linha 42, o termo “deteve” (significados “cessar, suspender, conter, refrear, reter”) não está de acordo com o sentido do texto. Sugiro usar “apresentou”.

Na parte de métodos, linhas 45-49, a informação desse “contexto” está repetitiva, pois foi descrita na introdução. Sugiro escolher onde a informação ficará, já que vem se repetindo várias vezes ao longo do texto.

Na página 3, linha 18, o termo “Regiões de Tratamento” pode dar o entendimento que se trata da parte corporal da pele. Sugiro usar “região (ou local) de atendimento do usuário”.

Não há justificativa clara para o uso da regressão de Poisson em vez de outras abordagens (exemplo: modelos logísticos para odds ratio). Uma breve explicação no texto sobre essa escolha seria útil.

Sugiro detalhar melhor o tempo entre o diagnóstico e tratamento para uma discussão mais aprofundada sobre a quantidade de pacientes que aguardam o tratamento além do tempo regulamentado. O prognóstico de quem conseguiu o tratamento em 31 dias é o mesmo de quem agaurdou 60 dias?

Deveria haver uma explicitação sobre possíveis limitações do estudo, como a qualidade dos dados secundários (erros de preenchimento, dados faltantes).

Na parte de resultados, na linha 32, não é necessário colocar o nome da tabela aqui no texto. Atentar para informações que são de resultados e estão na parte de discussão.

Na parte de discussão, o texto está repetitivo. Sugiro que a Tabela 2 seja melhor explorada na discussão, detalhando as razões de prevalência. Assim como as disparidades regionais que são apontadaspelo estudo, mas não são exploradas possíveis causas dessas diferenças (exemplo: infraestrutura hospitalar, distribuição de médicos especialistas).

